# Synthesis of Phosphated K-Carrageenan and Its Application for Flame-Retardant Waterborne Epoxy

**DOI:** 10.3390/polym10111268

**Published:** 2018-11-15

**Authors:** Na Wang, Haiwei Teng, Long Li, Jing Zhang, Ping Kang

**Affiliations:** 1 Sino-Spanish Advanced Materials Institute, Shenyang University of Chemical Technology, Shenyang 110142, China; 18842411721@163.com (H.T.); lilong@syuct.edu.cn (L.L.); zhangjingcszx@syuct.edu.cn (J.Z.); kangping@syuct.edu.cn (P.K.); 2 Advanced Manufacturing Institute of Polymer Industry (AMIPI), Shenyang University of Chemical Technology, Shenyang 110142, China

**Keywords:** waterborne epoxy, phosphated K-carrageenan, flame retardancy, DOPO

## Abstract

In this paper, phosphated K-carrageenan (P-KC) was obtained by reacting POCl_3_ with the renewable source K-carrageenan (KC). P-KC and 9,10-dihydro-9-oxa-10-phosphaphenanthrene-10-oxide (DOPO) were added into waterborne epoxy (EP) to improve its flame retardancy. The structure of P-KC was studied comprehensively using Fourier transform infrared spectroscopy (FTIR), X-ray photoelectron spectroscopy (XPS), thermo-gravimetric analysis (TGA), showing the successful synthesis of P-KC. The flame retardancy of the EP was evaluated by the cone calorimeter test. The results showed that different mass ratios of DOPO and P-KC affected the flame retardancy of EP. When the mass ratio of DOPO and P-KC was 2:1, total heat release (THR) and total smoke production (TSP) decreased by 48.7% and 37.4%, respectively. The microstructures of residue char were observed by FTIR and scanning electron microscopy (SEM), indicating that the flame-retardant waterborne epoxy (FR-EP) system held a more cohesive and denser char structure. The char inhibited the diffusion of heat and oxygen, which played a key role in the flame retardancy.

## 1. Introduction

Waterborne epoxy is widely used in steel structures and other fields due to its outstanding performance, in terms of its excellent chemical resistance to many chemical compounds and great adherence to various substrates [1,2]. However, waterborne epoxy is a flammable polymer material [3,4,5]. Flame retardants are additives that prevent polymer materials from being ignited or spread, and are widely used in the treatment of polymer materials [6,7]. Among them, intumescent flame retardant (IFR) is a halogen-free flame retardant, which has developed rapidly in recent years. When it is burned, IFR will form a dense layer of expanded char on the surface of a substrate; this char has an excellent ability to insulate the substrate from external heat and gas. Due to its high efficiency and the advantages of heat insulation, low smoke and low toxicity, IFR has become widely researched among flame retardants [8,9,10,11].

IFR is composed of an acid source, a carbon source, and a gas source. Among the carbon source of conventional IFR, polyhydroxy compounds produced by petroleum cracking are often used as a carbon source, such as pentaerythritol, phenolic resin, etc. [12,13,14,15]. However, with the depletion of petroleum resources and the pollution caused by petroleum use, it is necessary to find new carbon sources to replace petroleum [16,17]. Many biologically based materials with polyhydroxy structure are good carbon sources, such as starch [18,19], cellulose [20], tea saponin [21], cyclodextrin [22], carrageenan [23], etc., however, their thermal stability is not good. If the biologically based material is simply added to the matrix as a carbon source, the thermal stability of the matrix is lowered, and the char-forming ability is affected. According to the literature [24,25], the combination of phosphorus compounds (acid sources) and polysaccharide (char sources) is an effective approach to enhance the flame retardancy of composites.

This study aimed to enhance the thermal stability of natural materials and the char-forming ability in combustion. Phosphated K-carrageenan (P-KC) was obtained by the reaction of POCl_3_ with the natural substance K-carrageenan (KC). The structure and thermal stability of the as-prepared P-KC was characterized by Fourier-transform infrared spectroscopy (FTIR), X-ray photoelectron spectroscopy (XPS), and thermogravimetric analysis (TGA). We explored the effects of P-KC and 9, 10-dihydro-9-oxa-10-phosphaphenanthrene-10-oxide (DOPO) on the flame retardancy and thermal decomposition behavior of waterborne epoxy (EP), with different blending proportions of P-KC and DOPO.

## 2. Materials and Methods

### 2.1. Materials

KC was obtained from Zhejiang Jiaxing Maya Reagent Co., Ltd. (Jiaxing, China). Anhydrous ethanol, potassium chloride and pyridine were obtained from Tianjin Yongda Chemical Reagent Co., Ltd. (Tianjin, China). Hydrogen peroxide and analytical grade were obtained from Liaoning Jiacheng Fine Chemicals Co., Ltd. (Fuxin, China). Phosphorus oxychloride was obtained from Shandong West Asia Chemical Industry Co., Ltd. (Linyi, China). Formamide was obtained from Tianjin Fuchen Chemical Reagent Factory (Tianjin, China). Barium hydroxide was obtained from Tianjin Damao Chemical Reagent Factory (Tianjin, China). Acetone was obtained from Tianjin Dongfang Chemical Plant (Tianjin, China). Waterborne EP resin was obtained from Hexion Specialty Chemicals, Inc. (Columbus, OH, USA).

### 2.2. Methods

#### 2.2.1. Synthesis of P-KC

The KC was purified with 8% BaCl_2_ solution, and degraded with 6% hydrogen peroxide at 60 °C for 4 h, standing for one week. The pH value of the above solution was then adjusted to 7 with 1% NaOH solution, and the KC was washed three times with ethanol after being spun-dried, dried at 45 °C for 10 h lastly.

**Figure 1:**
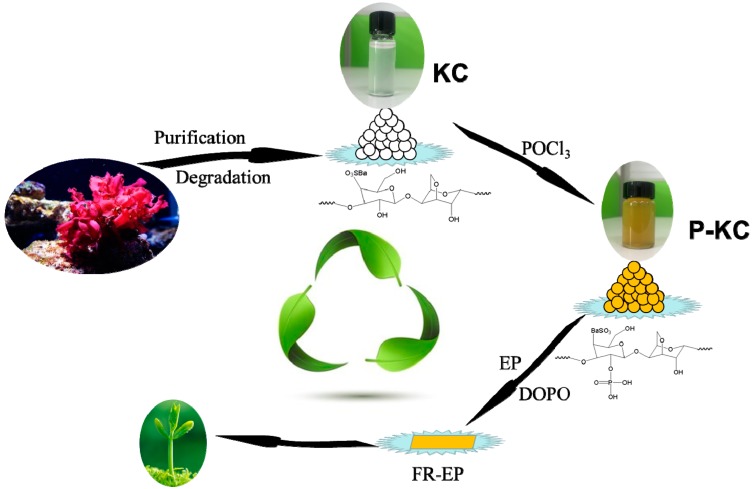
Synthetic route of K-carrageenan (KC) and phosphated K-carrageenan (P-KC), FR-EP.

The P-KC fillers were synthesized by our lab according to the literature [26]; the synthetic route is illustrated in Figure 1. A total of 30 mL of pyridine and 4 mL of POCl_3_ were added to a 250 mL three-necked flask, which was in an ice bath. After the addition, 2 g of the degraded KC and 15 mL of the formamide solution was dripped in batches, and the mixture was stirred throughout the whole addition. The reaction solution was poured into 150 mL of saturated Ba(OH)_2_ solution, the resulting brown precipitate was filtered, and the filtrate was poured into anhydrous ethanol and precipitated at room temperature overnight. The solution was centrifuged after standing, the supernatant was removed, and the precipitate was washed twice with anhydrous ethanol. Finally, a crude brown product was obtained. The crude product was dissolved in water, and the insoluble matter was removed after centrifugation. The supernatant was added to acetone and centrifuged again, and the resulting precipitate was washed three times with acetone and finally freeze-dried.

#### 2.2.2. Preparation of Flame-Retardant Coating

Based on extensive research by our group on flame retardants in polymers [27,28], this paper mainly explores the effect of different ratios of P-KC and DOPO on the flame retardancy of waterborne epoxy resin when added to 30 wt %. A series of flame-retardant coatings with different filler were prepared; the formulations are listed in Table 1. Taking the flame-retardant coating with 30 wt % P-KC as an example, P-KC (4.50 g) was blended with waterborne epoxy resin (7.50 g) using a pearl mill for 30 min, and curing agent and deionized water were added into the compound and stirred at room temperature for 15 min. The coating was scraped onto one side of a 100 × 100 × 1 mm^3^ steel plate, and the sample was cured at room temperature for one week before the test. As a comparison, other flame-retardant coatings of other filler were prepared by the same procedure.

#### 2.2.3. Characterization

The chemical structures of KC and P-KC were characterized by FTIR and XPS. FTIR spectra were recorded on a Nicolet MNGNA-IR560 (Artisan Technology Group, Austin, TX, USA) with thin films on KBr at room temperature. Transition mode was used and the wave-number range was set from 400 to 4000 cm^−1^. XPS spectra were obtained using a VG ESCALAB MKLL electron spectrometer (London, UK) equipped with an Al Ka X-ray source. Additionally, the thermal stability of KC and P-KC were characterized by STA 449C thermal analyzer (Selb, Germany), the measurement was in progress under N_2_ at a heating rate of 10 °C/min.

Limited oxygen index (LOI) data were obtained using an oxygen index instrument (JF-3) (Jiangning Analysis Instrument Company, Nanjing, China) according to GB/T 2406-2009 standard. The dimensions of the specimens were 126 × 6.5 × 3 mm^3^.

The vertical burning test was carried out on a CZF-3-type instrument (Jiangning Analysis Instrument Company, Nanjing, China) according to ASTM D3801-2010 standard. The dimensions of the specimens were 130 × 13 × 3 mm^3^ [29].

The cone calorimeter tests were carried out on a Fire Testing Technology (FTT, England, UK) cone calorimeter. The specimens were irradiated at a heat flux of 50 kW/m^2^ according to ISO 5660-1 standard procedures [30].

The SEM images of the burned samples were obtained with a JEOL JSM-6360LV scanning electron microscope (Hitachi, Tokyo, Japan). The burnt samples from LOI analysis were used for the testing. Complete burning of the samples was ensured.

## 3. Results and Discussion

### 3.1. Characterization of P-KC

#### 3.1.1. FTIR Results

**Figure 2:**
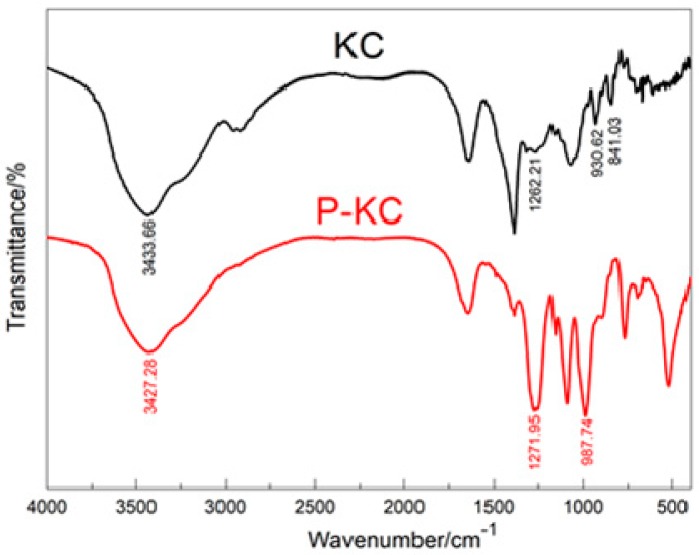
Fourier transform infrared spectroscopy (FTIR) spectra of KC and P-KC.

FTIR was used to confirm the preparation and characterization of P-KC. The FTIR spectra of KC and P-KC are shown in Figure 2: The peak at 1262.21 cm^−1^ resulted from the symmetric stretching vibration of O–S–O, indicating the presence of sulfate groups; the peak at 841.03 cm^−1^ resulted from the stretching vibration of C–O–S; and the stretching vibration of the C–O–C appeared at 930.62 cm^−1^. After reacting with POCl3, several new peaks appeared in the spectrum compared with that of KC. The peak at 1271.95 cm^−1^ could be ascribed to the stretching vibration of P=O, while the peak at 987.74 cm^−1^ could be assigned to the vibration absorption of P–O [31]. Thus, the reaction of KC and POCl_3_ was proven by the FTIR spectrum.

#### 3.1.2. XPS Test

**Figure 3:**
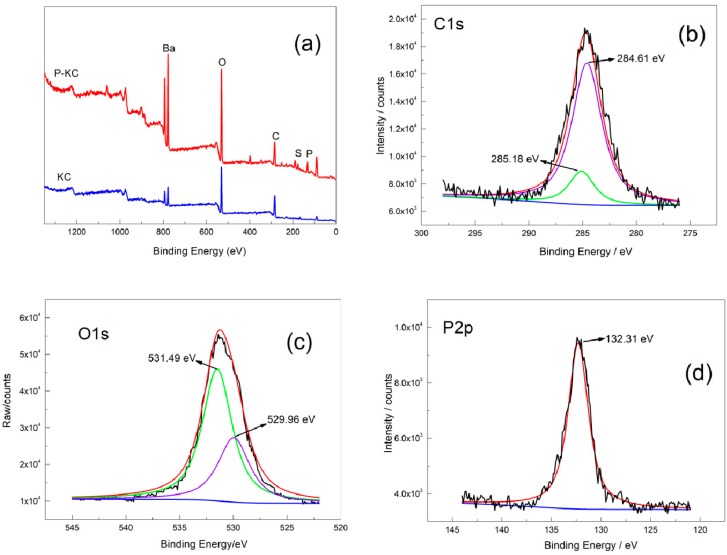
(**a**) X-ray photoelectron spectroscopy (XPS) full spectra of KC and P-KC. (**b**) C1s spectra of P-KC. (**c**) O1s spectra of P-KC. (**d**) P2p spectra of P-KC.

In order to further confirm the preparation of P-KC, XPS was used to analyze the varieties and states of different elements on the surface of P-KC. As can be seen in Figure 3a, KC and P-KC showed several main character peaks located at 780, 536, 292 and 173 eV, which were assigned to Ba3d, O1s, C1s and S2p signals, and which were consistent with FTIR result. Ba element was introduced by purifying KC with BaCl_2_. A phosphoric peak at 140 eV appears at the position of P-KC, while no phosphoric peak was detected for KC sample. C1s spectra fitting analysis (Figure 3b) showed the presence of C–O (285.18 eV), C–C, and C–H (284.61 eV) peaks [32]. O1s spectra fitting analysis (Figure 3c) showed the characteristic peak of oxygen in phosphate (531.49 eV) and –OH groups (529.96 eV) [33,34]. P2p spectral fitting analysis (Figure 3d) showed only one peak, the absorption peak of P–O (132.31 eV) [35].

Additionally, the surface chemical composition of the samples was measured by quantitatively calculating the atomic ratio of the P, S, C, and O elements. According to the atomic ratio of each element in the P-KC (Table 2), it can be calculated that the ratio between O1s (the oxygen element in the phosphate) and the P element was 4:1. Since a phosphorus atom was linked to four oxygen atoms in the phosphate molecule, it can be inferred that the P-KC was successfully synthesized [36].

#### 3.1.3. Thermogravimetric Analyses (TGA)

**Figure 4:**
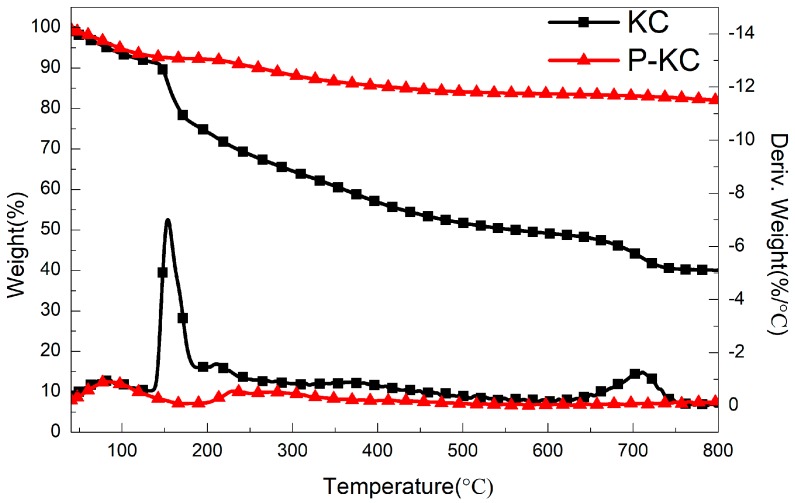
Thermogravimetric analysis and DTG curves of KC and P-KC under N_2_. (The top two lines belong to the left y-axis; the bottom two lines belong to the right y-axis).

Figure 4 presents the TGA and DTG curves of KC and P-KC under N_2_. As shown in the figure, the mass loss of KC below 150 °C was due to the thermal evaporation of bound water inside the molecule. KC decomposes in two steps at 150–700 °C, and the peak of heat release appears at 154 °C and 708 °C on the DTG curve. At 154 °C, mainly caused by glycosidic bond, six-membered ring decomposition and decarbonylation, the maximum heat release peak also occurs at 708 °C, which is due to decomposition of the unstable carbon layer, resulting in a char residual of 42 wt %. After the phosphating treatment of KC, P-KC only showed a small heat release peak at 230 °C. No weight loss occurred at high temperature, because the grafted phosphate ester was thermally decomposed to produce metaphosphoric acid and polyphosphoric acid during dehydration. KC forms a more stable char structure, which could slow down the transfer of heat and mass. The residual weight at 800 °C was increased to 80% compared with KC. The results show that only a few phosphorus groups were introduced into the structure of KC, and that P-KC achieves a good charring effect and thermal stability.

### 3.2. Thermal Stability of FR-EP

**Figure 5:**
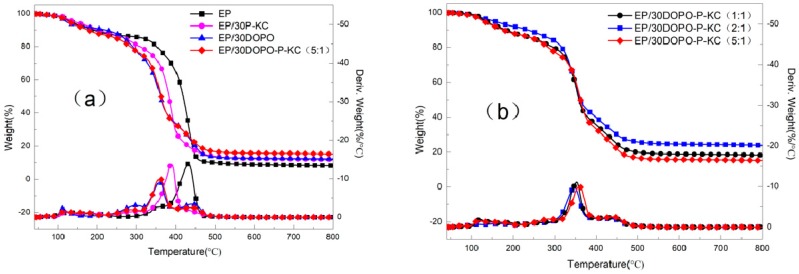
Thermogravimetric analysis and DTG curves of FR-EP under N_2_. (**a**) The top four lines belong to the left y-axis; the bottom four lines belong to the right y-axis; (**b**) the top three lines belong to the left y-axis; the bottom three lines belong to the right y-axis.

The thermal degradation results are shown in Table 3 and Figure 5a,b. The introduction of flame retardant reduces the thermal stability of the waterborne epoxy, mainly due to the catalytic effect of phosphoric acid produced by the phosphorus-containing flame-retardant system on the degradation of EP during the decomposition process [37,38]. *T*_20wt%_ of EP/30DOPO decreased with the addition of DOPO compared with pure EP (352 °C), and the residue at 800 Celsius increased. It can be seen from Figure 5a that with the addition of DOPO, a small thermal weight loss peak appeared at 300 °C, which possibly indicates the early decomposition of DOPO, and that DOPO catalyzed the char formation of EP and inhibited the decomposition of the matrix. When P-KC was added to EP alone, the *T*_20wt%_ of EP decreased, and C_800_ was improved compared with EP; this may be due to the early decomposition of P-KC in EP. As can be seen in Figure 5a, the addition of P-KC did not affect the charring behavior of EP, so P-KC could form char itself. When the mass ratio of DOPO and P-KC added to EP was 5:1, both *T*_20wt%_ and *T*_max%_ decreased, and the char residue of C_800_ was higher than the other three groups. This result may be due to DOPO and P-KC having a special reaction, because P-KC itself had many hydroxyl groups, which were easily esterified, the metaphosphoric acid and polyphosphoric acid formed by thermal decomposition of DOPO can react with these hydroxyl groups to form an esterification reaction, and thereby a stable char layer was formed which did not decompose at high temperature.

To optimize the ratio of DOPO to P-KC, the TGA and DTG characterization in N_2_ of EP/30DOPO-P-KC at different DOPO-P-KC ratios were performed; the results are shown in Figure 5b and Table 3.

As can be seen from Figure 5a, when the ratio of DOPO to P-KC was 2:1 or 1:1, the *T*_max_ was greatly reduced, and the amount of char residue of C_800_ was obviously increased. This may be due to an increase in the amount of P-KC, which produced enough char layer to protect the matrix.

### 3.3. Burning Behavior of FR-EP

#### 3.3.1. LOI and UL-94 Tests

In order to evaluate the flame retardancy of EP, LOI and UL-94 tests were performed; the relevant data are given in Table 4. It can be seen that the pure EP has a LOI of only 19.3%, UL-94 has no flame-retardant rating, and a large amount of droplets and smoke were generated during the combustion process. For EP/30P-KC, the addition of P-KC alone led to a barely detectable improvement in the flame retardation due to its low phosphorus content, which needed more acid to catalyze the char formation of the flame-retardant EP. When 30 wt % of DOPO was added alone, its UL-94 reached the V-1, but the value of LOI was still unsatisfactory. By adding DOPO and P-KC to the EP, the LOI value increased, and the vertical burning test also reached the V-1; this was due to the synergistic effect of DOPO and P-KC, which enhanced the flame-retardant properties of EP. When the total added amount of DOPO and P-KC were fixed at 30%, the ratio of P-KC to DOPO was changed, and the flame retardancy of EP changed as follows: In the flame-retardant performance, the ratio of DOPO to P-KC was 2:1, and it was better than the ratio of 1:1 and 5:1.

#### 3.3.2. Cone Tests

**Figure 6:**
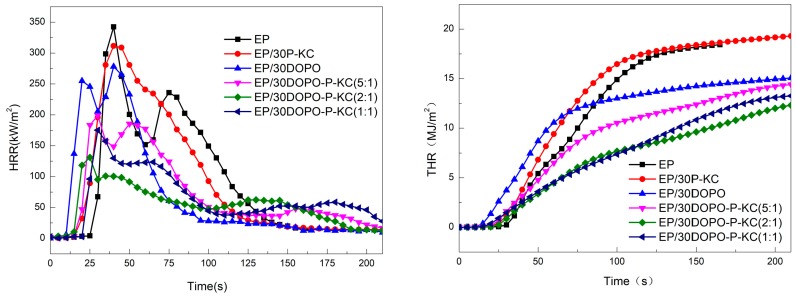
Heat release rate (HRR) curves of the pure waterborne epoxy (EP) and FR-EP.

The flame-retardant behavior of pure EP and its composites containing P-KC and DOPO was investigated by means of a cone calorimeter. The heat release rate and the total heat release versus time curves of composites are presented in Figure 6, and the detailed data are listed in Table 5. As can be seen, the total heat release (THR) and the peak of HRR (PHRR) of EP reached 18.3 MJ/m^2^ and 343.7 kW/m^2^, respectively. When DOPO was added alone, the THR and PHRR values decreased by 21% and 23%, respectively, whereas for EP/30P-KC, the PHRR value decreased, but the THR value increased to 19.3 MJ/m^2^ compared with the pure EP. This is in accordance with the results of the LOI and UL94 tests. After the loading of DOPO and P-KC together in EP, the THR and PHRR values of the EP decreased significantly. Additionally, the difference of heat release rate curves resulted from different ratios of DOPO and P-KC. For EP/30DOPO-P-KC (5:1), due to the excess DOPO, a polyphosphate char with cross-linked structure was formed. The expanding effect was restricted. When the temperature was raised again, the cross-linked polyphosphoric acid was thermally decomposed into P_4_O_10_ [39]. The thermal decomposition process of polyphosphoric acid caused the structurally intact expanded char layer to break, as for EP/30DOPO-P-KC (1:1), when the DOPO content is low, the protective char layer formed at the initial stage was not compact, and the flame retardancy of the expanded layer was lowered. However, the lowest THR and PHRR values of EP/30DOPO-P-KC (2:1) were 12.3 MJ/m^2^ and 131.0 kW/m^2^, respectively, indicating that the char layer formed at the initial time was quite compact and protected the underlying matrix very well. Therefore, when the mass ratio of DOPO to P-KC was controlled at 2:1, the combination of DOPO and P-KC had a good intumescent flame-retardant effect on EP, facilitating the formation of an efficient char barrier.

As can be seen from Table 5, the EP composite containing DOPO and P-KC largely has lower peak of CO production (PCOP) and total smoke production (TSP) values than pure EP. Because phosphoric acid was formed by thermal decomposition of FR-EP in the combustion process, it promotes char formation in the condensed phase, and the increase of char formation reduces the formation of small-molecule flammable gases by pyrolysis of the epoxy resin, indicating that the formed char layer plays a vital role in the flame-retardant and smoke-suppressing properties [40].

### 3.4. Structural Analysis of Combustion Residue

#### 3.4.1. FTIR Characterization

**Figure 7:**
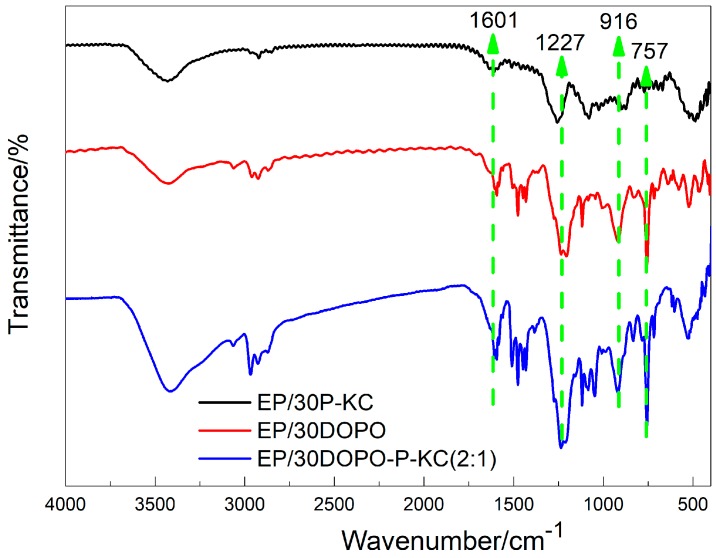
FTIR spectra of char from EP and FR-EP.

To study the mechanism of flame retardancy and the synergistic effect of the interactions between DOPO and P-KC, the char residues produced during the LOI test were analyzed by FTIR. As can be seen in Figure 7, the three groups all had a vibrational peak of the aromatic fused ring bone at 1601 cm^−1^; this was equivalent to improving the thermal stability of EP. However, EP/30DOPO-P-KC (2:1) showed several significant differences compared to EP/30DOPO and EP/30P-KC at 1227, 916, and 757 cm^−1^, which correspond to P=O, P–O, and P–C bonds, indicating that compounds containing more phosphorus were produced by DOPO and P-KC during combustion, and the char residue had a more stable high-temperature structure.

#### 3.4.2. SEM Analysis

**Figure 8:**
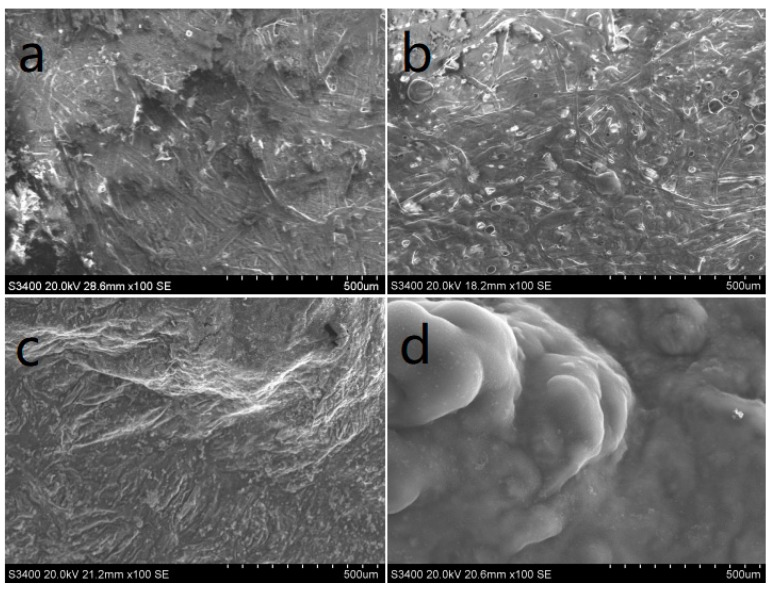
Scanning electron microscopy (SEM) micrographs of chars after LOI test. (**a**) EP; (**b**) EP/30DOPO; (**c**) EP/30P-KC; (**d**) EP/30DOPO-P-KC (2:1).

It is known that a continuous and expandable char layer can act as an insulating barrier to heat. In this study, SEM was used to investigate the char layer morphology of burnt EP composites obtained from the LOI test; the images are shown in Figure 8. It can be seen that the pure EP surface only formed a thin and discontinuous char layer with many defects, which had almost no flame retardancy. When DOPO was added to EP, the matrix was catalyzed to form a continuous char layer by DOPO, however the char layer was still thin with a few holes. The char layer for EP/30DOPO-P-KC (2:1) was denser and more swollen, and had a large number of wrinkles and protrusions compared with EP/30P-KC. This was because P-KC was catalyzed to form char during its dehydration, and the carbon layer obviously expanded, by the noncombustible gas CO_2_ and H_2_O. The surface of the char layer had wrinkles and protrusions, and the strength of the carbon layer was increased, so that the carbon layer was more easily maintained in the high-temperature airflow generated by combustion.

## 4. Conclusions

In this study, the biologically based flame-retardant P-KC was successfully synthesized. The results showed that the thermal stability and carbonization ability of P-KC were significantly improved due to the formation of phosphoric acid in situ during thermal decomposition. TGA data indicate that DOPO-decomposed phosphoric acid can react with the hydroxyl groups of P-KC; this was the cause of the improvement in thermal stability and char residue. According to the results of LOI and UL94, the optimal mass ratio of DOPO to P-KC was 2:1, the LOI of the EP/30DOPO-P-KC (2:1) reached 28.2% and UL94 reached V-0 grade. The cone test showed that THP, TSP, PCOP, release rate, and total smoke emission were reduced. The SEM and FTIR analysis showed that the carbon layer covering the surface of the substrate was dense and continuous. It can be concluded that the flame-retardant mechanism of DOPO and P-KC was mainly the flame retardancy of the condensed phase.

## Figures and Tables

**Table 1:** Formulations of coatings.

Sample	Waterborne Epoxy Resin/g	Curing Agent/g	P-KC/g	DOPO/g
Pure EP	10.71	4.29	—	—
EP/30DOPO	7.50	3.00	—	4.50
EP/30P-KC	7.50	3.00	4.50	—
EP/30DOPO-P-KC (5:1)	7.50	3.00	0.75	3.75
EP/30DOPO-P-KC (2:1)	7.50	3.00	1.50	3.00
EP/30DOPO-P-KC (1:1)	7.50	3.00	2.25	2.25

**Table 2:** Electron binding energy and atomic ratio of main elements on P-KC surface.

Element	Peak Position (eV)	Atomic Ratio (%)
P	132.31	9.31
S	166.62	1.33
C	284.61	27.37
	285.18	5.44
O	529.96	19.30
	531.49	37.25

**Table 3:** Thermo-gravimetric analysis (TGA) and DTG data of FR-EP in N_2_ atmosphere.

Sample	*T*_20wt%_ (°C)	*T*_max_ (°C)	C_800_ (%)
EP	352	432	8.1
EP/30P-KC	309	388	11.8
EP/30DOPO	290	360	12.5
EP/30DOPO-P-KC (1:1)	297	352	18.3
EP/30DOPO-P-KC (2:1)	317	346	23.8
EP/30DOPO-P-KC (5:1)	283	359	15.2

**Table 4:** Limited oxygen index (LOI) and UL-94 test results of different samples.

Sample	LOI/%	UL-94
EP	19.3	No rating
EP/30DOPO	22.1	V-1
EP/30P-KC	20.8	No rating
EP/30DOPO/P-KC (5:1)	25.0	V-1
EP/30DOPO/P-KC (2:1)	28.2	V-0
EP/30DOPO/P-KC (1:1)	27.1	V-0

**Table 5:** Cone calorimetry data for the pure EP and FR-EP.

Sample	PHRR (kW/m^2^)	THR (MJ/m^2^)	PCOP (g/s)	TSP (m^2^/kg)
EP	343.7	18.3	0.0162	5.2946
EP/30P-KC	313.7	19.3	0.0071	4.7162
EP/30DOPO	279.6	15.1	0.0064	4.5553
EP/30DOPO-P-KC (1:1)	176.4	13.3	0.0052	4.2223
EP/30DOPO-P-KC (2:1)	131.0	12.3	0.0050	3.8513
EP/30DOPO-P-KC (5:1)	197.0	14.5	0.0058	4.0491
